# Design Your Clinical Workplace to Facilitate Competency-Based Education

**DOI:** 10.5811/westjem.2019.4.43216

**Published:** 2019-06-11

**Authors:** Holly A. Caretta-Weyer, Michael A. Gisondi

**Affiliations:** Stanford University School of Medicine, Department of Emergency Medicine, Palo Alto, California

Competency-based education (CBE) is a criterion-referenced, outcomes-based framework used for curriculum design and assessment in medical education.^1^ The goals of CBE are to define and assess provider competence along a trajectory, from novice to expert, using objective performance measures.^2^

CBE requires five key design features to be successful.^3^ (1) The first requirement is an outcomes-based competency framework. The Accreditation Council for Graduate Medical Education (ACGME) developed the ACGME Milestones Project to operationalize competency domains for postgraduate trainees.^4^ (2) The second expectation is that competencies and their developmental markers must support learner progression along a continuum. The Milestones represent just such a developmental model, with defined behavioral outcomes for residents as they progress through training. The remaining key features of CBE include (3) individually-tailored learning experiences within an authentic workplace environment that are sequenced or designed in response to each resident’s developmental trajectory; (4) coaching for individual resident growth and achievement; and (5) programmatic assessment using multiple methods of data collection. High-quality data is sourced from numerous assessors, at a high frequency, mapped to the chosen framework, and used for robust summative entrustment and promotion decision-making by a clinical competency committee (CCC).^5^–^6^ This approach to assessment is labor-intensive but results in publicly-defensible decisions about the training and competence of our physician workforce.

## The Challenges of Competency-based Education in Emergency Medicine

The ACGME Milestones can be operationalized to meet each of the CBE design features listed above. However, Sheng et al. identified significant challenges in their local implementation and utilization of this framework.^7^ The true intent of the ACGME Milestones is to represent the developmental trajectory of a resident over time. The variability in faculty ratings, which may initially seem frustrating, are instead intentionally important to the process of monitoring development in our trainees. There is no perfect assessment tool and there will be variability in assessments. And that’s okay.

The attainment of competence is rarely a linear path for a resident, just as developmental progression is not the same for children.^5^,^8^ Residents learn at different rates, have different strengths, and different areas for improvement. These areas for improvement are rarely stable throughout three or four years of residency training. The lack of inter-rater consistency among faculty is normal and expected. The beauty of the Milestones is that the vast array of “dots” on the submission form – or assessment data points – will eventually provide a general understanding of each resident’s trajectory. The greater number of data points, the clearer the trajectory, especially if data points are sourced from a wide range of supervisory perspectives.

“Does context matter?” The answer is clearly yes, for each separate data point. It is thus up to the trained group of faculty members who comprise the CCC to interpret those data and the context from which they arose. The Committee may then provide an *educated decision* about a trainee’s progress and propose an individually-tailored learning plan to address any areas of deficiency. An educated decision is *not a random guess* if the decision is based on adequate data; if there are inadequate data, then your CCC has simply identified an *assessment gap* for which the program needs to obtain more or better data.^9^

Optimizing this process is difficult without first changing our approach to obtaining Milestones data. Aggregate evaluations at the end of each rotation generally lack the necessary timeliness, granularity, or approachability to provide learners with truly actionable feedback.^10^ Residents often do not understand the Milestones themselves and simply look to any narrative comments on their evaluations to obtain insight into their performance.^11^ We need to rethink our process of data collection and separate that process from decisions about the Milestones.

CBE is successful when there is a substantial emphasis on workplace-based assessment of trainees.^5^,^12^ True workplace-based assessment requires at least some direct observation.^5^,^11^ Unfortunately, there are abundant barriers to conducting planned observations in our workplaces. Emergency departments (ED) are fraught with interruptions, high volumes of patients, and competing priorities for faculty members’ attention. These practice realities can distract faculty members and trainees from seeking opportunities for lengthy, formal observations during a clinical shift.

Failure to optimize workplace-based assessments in the ED is often blamed on the complexity of the Milestones rather than the workplace itself. We can design impressive assessments, but if our learning environments have barriers to performing observations or providing timely feedback, these assessments may be rendered useless to all stakeholders. The inability to obtain quality data about our learners may result from misalignment of our assessment methods and our workplaces. This lack of data ultimately impairs the work of the CCC, which leads to suboptimal group processes, uninformed promotion decisions, and a poor functional understanding of CBE by our residency core faculty members.

## Strategies for Success

The ED can be deliberately designed to facilitate CBE. Emergency medicine faculty members make frequent subconscious decisions about entrustment and trainee supervision throughout each shift. We regularly allow residents to perform procedures with on-demand supervision, or we step back to observe senior residents as they direct a resuscitation team. Simply “recording” these moments and providing direct feedback to trainees operationalizes workplace-based assessment without necessitating the use of the Milestones in real time. It is the responsibility of the CCC to map the documentation of these assessments back to the Milestones for aggregate decision-making and reporting purposes. Such frontline documentation does not need to include any Milestones language. We do not need to train every faculty member to deeply understand the Milestones framework; rather we need to train our CCC members to interpret available data.^13^–^14^

Desired outcomes must drive assessments. If you want your senior residents to perform a tube thoracostomy with only indirect supervision, then you need to optimize the learning environment to ensure adequate opportunities for them to practice this task. Learning outcomes are workplace-based *tasks*; outcomes are not the Milestones themselves. If your workplace environment does not support your current assessment methods, redesign the workplace or change the assessment method. Augment data collection with other modalities such as multisource feedback and simulation. Then design faculty development and resident training that follows your redesigned assessment process.

Faculty development is one of the most frequently identified challenges to assessment, particularly as it relates to the Milestones.^15^ Many faculty development challenges may be ameliorated by simplifying frontline assessments to more intuitive forms that use task-based evaluations and supervisory language. Data from these forms can then be mapped back to the Milestones by the CCC. The Milestones were not designed to be copied onto evaluation forms directly as they are written; they were also not designed for use by frontline faculty.^16^,^17^ By making workplace assessment tools more usable by the general faculty, you minimize your need for intensive faculty development. Use digital assessment tools rather than paper, if available; smartphone apps are more convenient than desktop portals for frontline evaluations. In addition, encourage residents to seek feedback using simply designed, self-assessment tools. These changes will reduce the burden of day-to-day assessment for faculty members, creating a shared responsibility to generate feedback for both faculty members and residents.

The value of the CCC can be maximized through better group processes for summative decision-making. The guiding purpose of the CCC may in fact be twofold: to make decisions about each resident’s progress using the Milestones, and to critically appraise the assessment data available to the committee on a regular basis.^18^ Identify gaps in assessment that are preventing the CCC from making informed decisions about learner development and progression. Use available data to develop individualized learning plans and coach the residents accordingly.

To ensure that CCC decisions are defensible and accurate, there must be a structured format for committee meetings so that all stakeholder viewpoints are represented. This is often best accomplished by a having faculty “expert” for each postgraduate year (PGY) class.^13^,^18^ Involve each CCC member in the pre-review process of trainees in their assigned PGY class and allow them to lead the discussion of these trainees during the meetings. Provide CCC members with early access to high-quality assessment data and conduct regular training of the CCC members. Finally, stakeholder output is a key mandate of the CCC. While Milestone ratings are the necessary administrative outcome, individualized learning plans may be the more important CCC goal.^18^ These learning plans should document areas for improvement to be reviewed with trainees by their assigned faculty coaches.

## SUMMARY

Challenge your residency leadership and CCC to design a workplace that facilitates CBE. Create simpler frontline assessments that avoid the direct use of Milestone language and supplement with multisource feedback and simulation to address assessment gaps. Map frontline assessment tools to the Milestones behind the scenes. Be deliberate about what you intend to assess, ensuring that assessment reflects key learning outcomes of the curriculum. Schedule time for direct observation of learners. Use faculty development and resident training to create a shared understanding of CBE and its application in your ED. Resolve any disconnect between obtaining data and using data. Empower residents to seek feedback during clinical shifts, to review self-assessments with their faculty coaches, and to fully engage in the assessment process at your institution. Finally, focus the outcomes of CCC meetings not simply on the assignment of Milestones, but on the creation of individual learning plans that promote trainee development.
